# Effective alkaline metal-catalyzed oxidative delignification of hybrid poplar

**DOI:** 10.1186/s13068-016-0442-0

**Published:** 2016-02-09

**Authors:** Aditya Bhalla, Namita Bansal, Ryan J. Stoklosa, Mackenzie Fountain, John Ralph, David B. Hodge, Eric L. Hegg

**Affiliations:** DOE Great Lakes Bioenergy Research Center, Michigan State University, East Lansing, USA; Department of Biochemistry and Molecular Biology, Michigan State University, East Lansing, USA; Department of Chemical Engineering and Materials Science, Michigan State University, East Lansing, USA; DOE Great Lakes Bioenergy Research Center, University of Wisconsin-Madison, Madison, USA; Division of Sustainable Process Engineering, Luleå University of Technology, Luleå, Sweden

**Keywords:** Alkaline hydrogen peroxide (AHP) pretreatment, Biomass conversion, Catalysis, Cellulosic biofuels, Copper, Hybrid poplar, Lignin, Oxidative delignification, Sugars

## Abstract

**Background:**

Strategies to improve copper-catalyzed alkaline hydrogen peroxide (Cu-AHP) pretreatment of hybrid poplar were investigated. These improvements included a combination of increasing hydrolysis yields, while simultaneously decreasing process inputs through (i) more efficient utilization of H_2_O_2_ and (ii) the addition of an alkaline extraction step prior to the metal-catalyzed AHP pretreatment. We hypothesized that utilizing this improved process could substantially lower the chemical inputs needed during pretreatment.

**Results:**

Hybrid poplar was pretreated utilizing a modified process in which an alkaline extraction step was incorporated prior to the Cu-AHP treatment step and H_2_O_2_ was added batch-wise over the course of 10 h. Our results revealed that the alkaline pre-extraction step improved both lignin and xylan solubilization, which ultimately led to improved glucose (86 %) and xylose (95 %) yields following enzymatic hydrolysis. An increase in the lignin solubilization was also observed with fed-batch H_2_O_2_ addition relative to batch-only addition, which again resulted in increased glucose and xylose yields (77 and 93 % versus 63 and 74 %, respectively). Importantly, combining these strategies led to significantly improved sugar yields (96 % glucose and 94 % xylose) following enzymatic hydrolysis. In addition, we found that we could substantially lower the chemical inputs (enzyme, H_2_O_2_, and catalyst), while still maintaining high product yields utilizing the improved Cu-AHP process. This pretreatment also provided a relatively pure lignin stream consisting of ≥90 % Klason lignin and only 3 % xylan and 2 % ash following precipitation. Two-dimensional heteronuclear single-quantum coherence (2D HSQC) NMR and size-exclusion chromatography demonstrated that the solubilized lignin was high molecular weight (M_w_ ≈ 22,000 Da) and only slightly oxidized relative to lignin from untreated poplar.

**Conclusions:**

This study demonstrated that the fed-batch, two-stage Cu-AHP pretreatment process was effective in pretreating hybrid poplar for its conversion into fermentable sugars. Results showed sugar yields near the theoretical maximum were achieved from enzymatically hydrolyzed hybrid poplar by incorporating an alkaline extraction step prior to pretreatment and by efficiently utilizing H_2_O_2_ during the Cu-AHP process. Significantly, this study reports high sugar yields from woody biomass treated with an AHP pretreatment under mild reaction conditions.

**Electronic supplementary material:**

The online version of this article (doi:10.1186/s13068-016-0442-0) contains supplementary material, which is available to authorized users.

## Background

Sustainably produced lignocellulosic biomass is a promising feedstock for the production of petroleum-displacing liquid transportation fuels [1, 2]. Structural polysaccharides (i.e., cellulose and hemicelluloses) comprise the majority of the mass of plants and are amenable to biochemical and/or catalytic conversion, serving as non-food alternatives to starch- and sucrose-derived biofuels [2]. However, plant cell walls exhibit recalcitrance to these approaches to deconstruction and conversion due to a variety of cell wall properties that include higher order structures across multiple length scales [3]. Foremost among these features contributing to cell wall recalcitrance is lignin, which limits access of hydrolytic enzymes to these fermentable sugars [4].

Relative to herbaceous biomass, woody biomass has attractive features as a feedstock for the production of renewable fuels and chemicals due to its higher bulk density and year-round availability that facilitates feedstock logistics [5, 6]. Unfortunately, woody biomass can also be substantially more recalcitrant to deconstruction and conversion than graminaceous feedstocks (i.e., grasses) due, in part, to the high lignin content [7]. For deconstruction routes employing enzymatic hydrolysis, pretreatment is an essential step necessary to remove, modify, and/or redistribute the lignin [8, 9]. Many of the existing reported pretreatment approaches for hardwoods, however, are capital intensive and require high temperature and/or pressure [3, 10–12].

Delignifying alkaline pretreatments (as well as alkaline pulping technologies) overcome cell wall recalcitrance by chemically modifying and/or cleaving lignin to increase its solubility in alkali [13, 14]. Furthermore, depending on the conditions used for pretreatment, a substantial fraction of the hemicelluloses may also be solubilized and potentially degraded [15]. Therefore, in addition to improving biomass enzymatic digestibility by removing lignin, alkaline pretreatments can also be considered as biomass fractionation processes that have the capacity to provide a separate lignin stream that can be valorized to additional coproducts [13, 16, 17].

Alkaline-oxidative pretreatments such as alkaline hydrogen peroxide (AHP) have been shown to be effective pretreatments for herbaceous feedstocks [18–23] as well as for woody biomass [24–26], although these approaches have often employed prohibitively high oxidant loadings on the biomass (e.g., 250 to greater than 2000 mg H_2_O_2_ per g biomass). We recently discovered that adding small amounts of copper 2,2′-bipyridine complexes [Cu(bpy)] during AHP pretreatment (Cu-AHP) resulted in substantially improved sugar yields following enzymatic hydrolysis relative to AHP pretreatment in the absence of the copper catalyst (AHP-only) at modest oxidant loadings (e.g., 25 to 100 mg H_2_O_2_ per g biomass) [27, 28].

In this manuscript, we describe a combination of strategies to increase the effectiveness of Cu-AHP as well as reduce the chemical inputs to improve the process economics. These enhancements include (i) the addition of an alkaline extraction step prior to Cu-AHP pretreatment and (ii) the more efficient utilization of H_2_O_2_. Together these modifications substantially increased lignin and xylan removal, which resulted in significantly improved sugar yields following enzymatic hydrolysis. In addition, this modified process also yielded relatively pure and unmodified lignin compatible with subsequent valorization.

## Results and discussion

### Fed-batch addition of H_2_O_2_

**Fig. 1:**
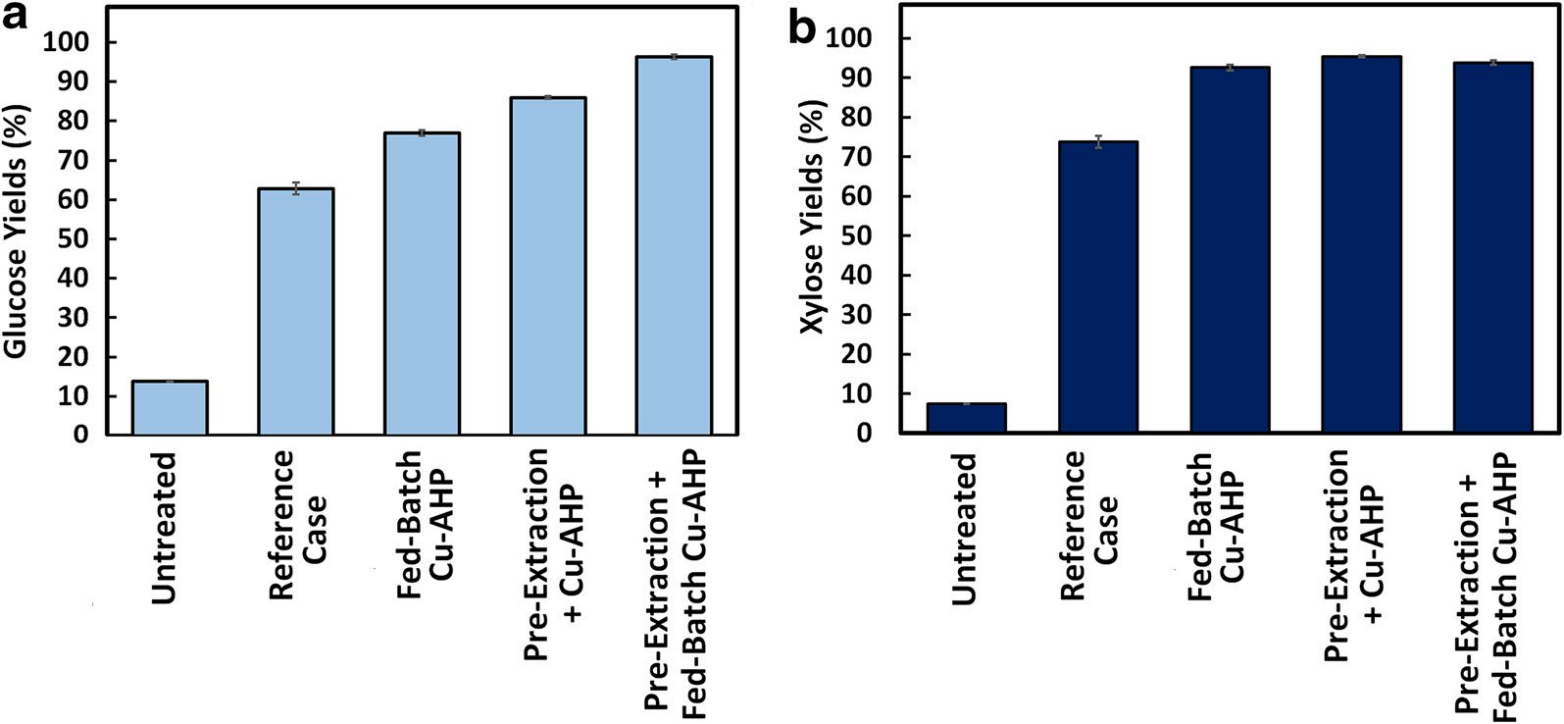
Glucose (**a**) and xylose (**b**) yields obtained following enzymatic hydrolysis of hybrid poplar pretreated under our standard (reference case) or our modified Cu-AHP conditions. Fed-batch Cu-AHP indicates fed-batch addition of H_2_O_2_ and pre-extraction refers to alkaline pre-extraction prior to Cu-AHP pretreatment. The points are the averages of three biological replicates, and error bars indicate ± standard deviations of the means

Hydrogen peroxide represents an important contribution of the cost associated with AHP pretreatment [19, 29], and identifying strategies to reduce the H_2_O_2_ loading without compromising sugar yields would therefore lead to substantial improvements in the operating costs. H_2_O_2_ spontaneously disproportionates to O_2_ and water in a concentration-dependent reaction under alkaline conditions near its p*K*_a_ of 11.5 [21]. We hypothesized that by lowering the effective H_2_O_2_ concentration during the reaction, these non-productive reactions might be decreased, enabling more of the reactive oxygen species to react with the biomass. To test this hypothesis, we added the H_2_O_2_ in small batches during the course of the pretreatment without changing either the total amount of oxidant utilized or the pretreatment time. Relative to the reference case where all of the H_2_O_2_ was added at the beginning of the pretreatment, this modification resulted in a ~1.2 fold increase in sugar yields (77 % glucose and 93 % xylose) following enzymatic hydrolysis (Fig. 1; Additional file 1: Table S1). This strategy has also been reported in the literature where H_2_O_2_ was added in a fed-batch manner to avoid its unproductive decomposition during epoxidation [30–32] and hydroxylation [33–35] reactions.

**Fig. 2:**
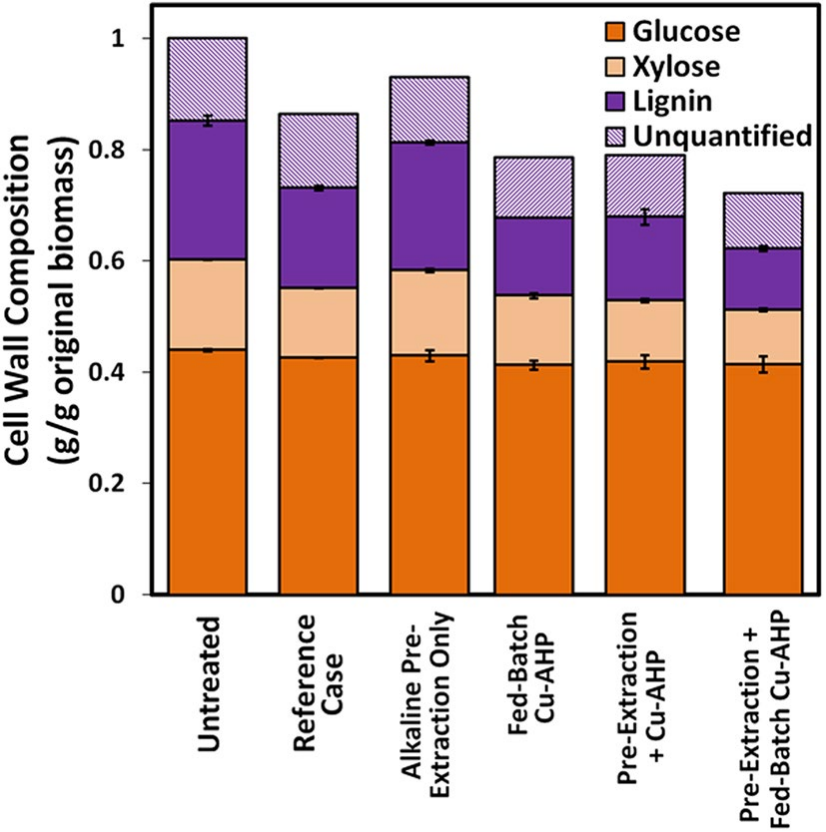
Mass loss and cell wall composition change associated with pretreatments under standard (reference case) or modified Cu-AHP conditions. Fed-batch Cu-AHP indicates fed-batch addition of H_2_O_2_ and pre-extraction refers to alkaline pre-extraction prior to Cu-AHP pretreatment. The values reported are the averages of the three biological replicates, and the error bars indicate ± standard deviations of the means

To ascertain how fed-batch addition of H_2_O_2_ alters the cell wall composition of hybrid poplar relative to the reference case, compositional analysis of the biomass was performed both before and after pretreatment. The results revealed that the major impact of fed-batch addition of H_2_O_2_ was an increase in the extent of lignin removal, with approximately 44 % of the original lignin solubilized relative to only 28 % for the reference case (Fig. 2; Additional file 1: Table S2). Delignification is well known to improve enzymatic hydrolysis sugar yields by improving hydrolytic enzymes’ access to the cellulose [15, 36, 37].

### Two-stage pretreatment employing alkaline pre-extraction

Seeking to increase further the efficacy of the pretreatment process, we incorporated an alkaline extraction step prior to the Cu-AHP pretreatment. The rationale was that removing easily extracted hemicellulose and lignin might improve penetration of the Cu(bpy) complexes into the plant cell wall, thereby leading to more effective targeting of the active radical species to unextracted cell wall lignin. In addition, this step would also remove extractives and easily solubilized lignin, potentially reducing the inhibition of enzymes during hydrolysis. This strategy is comparable to coupling alkaline delignification with an oxidative post-treatment utilized in the established processes for chemical pulping and oxidative bleaching or delignification of wood in the pulp and paper industry [38]. Liu et al. [39] previously employed a similar approach with corn stover, coupling an alkaline pre-extraction with an AHP post-treatment at only 25 mg H_2_O_2_/g biomass, and they achieved near-theoretical glucose yields following hydrolysis at modest enzyme loadings. In addition, Koo et al. [40] and Draude et al. [41] demonstrated improved sugar yields utilizing alkaline pretreatment followed by oxygen delignification as a post pretreatment. Finally, Yuan et al. [42] demonstrated effective and synergistic lignin and hemicelluloses removal from poplar using a two-step alkaline and ionic liquid pretreatment. As a further benefit, alkaline pre-extraction can be performed on wood chips [15], which subsequently reduces the energy required for effective comminution relative to non-pre-extracted biomass.

Based on the final sugar yields (Fig. 1), the alkaline pre-extraction step had a large positive impact on enzymatic hydrolysis yields. Relative to the single-stage reference case, the addition of the alkaline pre-extraction step improved glucose yields from 63 to 86 % and xylose yields from 74 to 95 % (Fig. 1; Additional file 1: Table S1). Compositional analysis following alkaline pre-extraction of hybrid poplar indicated that whereas ~5 % of both the lignin and xylan was solubilized during pre-extraction (Fig. 2; Additional file 1: Table S2), essentially none of the glucose was extracted. It should be noted that under the mild alkaline reaction conditions employed in this study, the xylan should be extensively deacetylated, which aids saccharification, but otherwise be relatively undegraded with a high potential for recovery. Cu-AHP pretreatment of this alkaline pre-extracted poplar resulted in the solubilization of 40 % of the original lignin, which is about 1.4 times higher than in the reference case. Importantly, synergy was observed in the two-stage pretreatment approach, with the combined alkaline pre-extraction/Cu-AHP approach removing nearly 8 % more lignin than the sum of either of the two stages alone. This increased delignification is consistent with the significant increase in sugar yields following enzymatic hydrolysis for the combined two-stage approach.

Hypothesizing that alkaline pre-extraction aids pretreatment, in part, by removing easily extractable lignin, thereby increasing the surface area for easy penetration of metal complexes during pretreatment, we measured the water retention value (WRV) both before and after pre-extraction. Our results indicated that the WRV of hybrid poplar increased from 1.15 g water/g biomass (untreated) to 1.50 g water/g biomass following the alkaline pre-extraction step (Additional file 1: Figure S1), consistent with the importance of WRV to the pretreatment process. The swelling of biomass, which increases the internal surface area (as indicated by the WRV), has been observed previously following pretreatment with alkali using NaOH, KOH, or Ca(OH)_2_ [43–45].

To ascertain if alkaline pre-extraction also reduced enzyme inhibition by removing extractives and lignin, we evaluated the effect of pre-extraction liquor on the hydrolysis of a model crystalline cellulose substrate (Avicel PH 101). The results demonstrated that enzymatic hydrolysis of Avicel in the presence of alkaline pre-extraction liquor resulted in a 6 % decrease in the glucose yields compared to hydrolysis in buffer only (Additional file 1: Figure S2). It should be noted that this difference was observed under high enzyme loadings (30 mg/g glucan) and long incubation times (24 h), and this effect would likely be magnified at low enzyme loadings and shorter enzymatic hydrolysis times. Together, this result suggests that in addition to biomass swelling, pre-extraction of the hybrid poplar with NaOH increases sugar yields, at least in part, by removing alkali-extractable compounds that inhibit enzymatic hydrolysis.

### Combining alkaline pre-extraction and fed-batch H_2_O_2_ addition

Having demonstrated that the efficacy of Cu-AHP could be improved dramatically by fed-batch addition of the H_2_O_2_ over the course of the pretreatment as well as by extracting the poplar with alkali prior to Cu-AHP (Fig. 1), we next sought to ascertain if the two strategies could be combined to improve even further the sugar yields. Combining these two strategies resulted in the solubilization of nearly 40 % of the xylan and over 55 % of the lignin during pretreatment (Fig. 2; Additional file 1: Table S2), a substantial improvement over the reference case that did not include either of these modifications. As expected, this increase in lignin and xylan (hemicellulose) removal resulted in a significant improvement in sugar yields (Fig. 1). In fact, enzymatic hydrolysis resulted in high sugar yields of 96 and 94 % of the theoretical maximum (based on original composition) for glucose and xylose, respectively. Importantly, xylan solubilization did not result in decreases in the xylose yields because the xylan was deacetylated but not degraded and therefore remained available in the pretreatment mixture for enzymatic hydrolysis. It is also important to note that only a small quantity of xylose oligomers was released and subsequently lost during alkaline pre-extraction compared to the xylose oligomers released during the following Cu-AHP step (Fig. 1b).

Several previous studies have examined the effects of a variety of pretreatments on the conversion of hardwoods, and a few have reported high sugar yields [10, 11, 36, 42, 46–48]. The pretreatment strategy described herein is performed at atmospheric pressure, and as a result, it can be performed without the added capital costs associated with high pressure and high-temperature reactors. High sugar yields from hybrid poplar have also been reported utilizing metal-catalyzed AHP under standard atmospheric conditions, although these reactions required significantly greater H_2_O_2_ loadings (~1000 mg/g biomass) to obtain comparable sugar yields [26, 49]. To the best of our knowledge, this is the first study that reports high sugar yields (i.e., >95 %) from a woody biomass treated with an AHP pretreatment with low H_2_O_2_ loadings under mild reaction conditions (i.e., low temperature and pressure).

### Impact of enzyme, H_2_O_2_, and catalyst loadings on fed-batch, two-stage Cu-AHP pretreatment

**Fig. 3:**
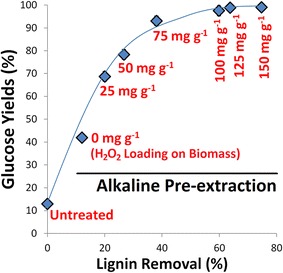
Correlating glucose yields following enzymatic hydrolysis of alkaline pre-extracted + fed-batch Cu-AHP pretreated hybrid poplar with lignin removal at different H_2_O_2_ loadings. The points are the averages of three separate experiments, and the error bars indicate ± standard deviations of the means

Next, a series of experiments were performed to explore the relationship between pretreatment conditions and the biomass response. Specifically, the impacts of H_2_O_2_ and catalyst loading during Cu-AHP pretreatment as well as enzyme loading during enzymatic hydrolysis were investigated with the goals of (1) ascertaining how these variables impact hydrolysis yields and (2) identifying strategies for how these inputs into the process may be minimized, while maintaining high hydrolysis yields. In the first set of experiments, the H_2_O_2_ loading was varied for alkaline pre-extracted biomass while holding the other variables constant, and the impact on lignin removal and hydrolysis yields was determined (Fig. 3). For the reaction conditions used, the results demonstrated that the H_2_O_2_ loading can be reduced by 25 to 75 mg/g poplar while still maintaining sugar yields over 90 %. In fact, even at H_2_O_2_ loadings as low as 50 mg H_2_O_2_/g biomass, the sugar yields following enzymatic hydrolysis were higher (78 %) than those obtained in the reference case (63 %) which utilized 100 mg H_2_O_2_/g biomass. As expected, compositional analysis of the biomass treated with different H_2_O_2_ loadings revealed a clear relationship between the extent of lignin removal and the sugar yields following enzymatic hydrolysis (Fig. 3). Sugar yields increased rapidly with lignin removal until approximately 40 % of the lignin had been removed (to ~14 % final lignin content in the pretreated biomass) at which point additional lignin removal had only a modest effect on sugar yields. A similar threshold value for hydrolysis yields between a Klason lignin content of 10–15 % has been observed by Grabber et al. [50] and Li et al. [44] for grasses subjected to delignification.

**Fig. 4:**
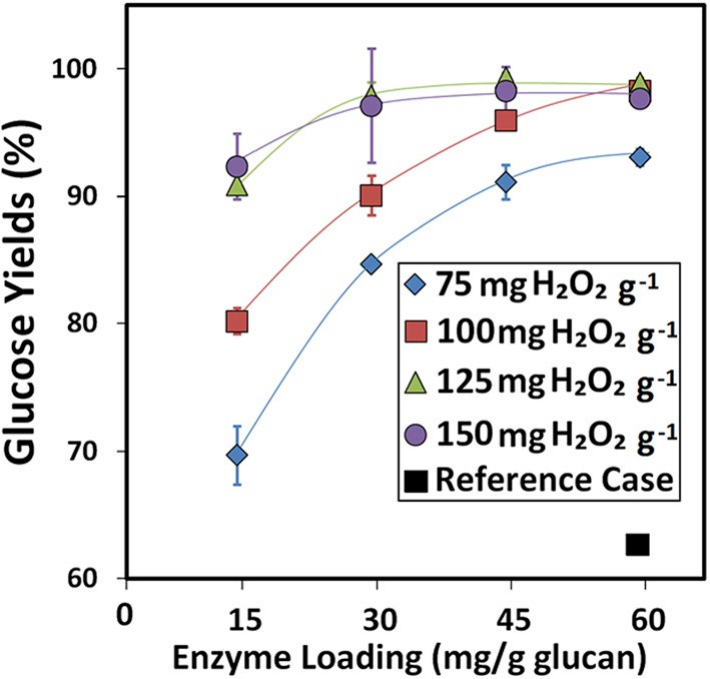
Impact of enzyme loading and H_2_O_2_ loading on hydrolysis yields of glucose following both alkaline pre-extraction and Cu-AHP pretreatment utilizing fed-batch addition of H_2_O_2_. The data points are the averages of three independent experiments, and the error bars indicate ± standard deviations of the means

Enzymes currently represent an important operating cost in essentially all cellulosic biofuels processes employing an enzymatic deconstruction step [51, 52]. Consequently, there has been considerable focus on improving pretreatment strategies to enable lower enzyme loadings to be used during hydrolysis without sacrificing sugar yields [53]. The first set of experiments described above was performed utilizing high enzyme loadings of 60 mg protein (enzyme) per g glucan. Hypothesizing that the alkaline pre-extraction and the more efficient utilization of H_2_O_2_ might allow us to reduce enzyme loading, we initiated a second set of experiments to correlate glucose yields following enzymatic hydrolysis of pretreated hybrid poplar using different enzyme loadings (Fig. 4). Not surprisingly, there was a strong correlation between enzyme loading and sugar yield. As anticipated, however, alkaline pre-extraction coupled with fed-batch addition of H_2_O_2_ during Cu-AHP treatment resulted in improved sugar yields even at much lower enzyme concentrations. For instance, at 100 mg H_2_O_2_ per gram poplar (10 % H_2_O_2_), we were able to reduce enzyme loadings to 15 mg/g glucan (a fourfold decrease) and still obtain significantly higher glucose yields following enzymatic hydrolysis (approximately 80 versus 60 % for the Cu-AHP reference case conditions). In addition, when H_2_O_2_ loadings were increased to 125 mg H_2_O_2_/g biomass, glucose yields increased still further to greater than 90 % while utilizing only 15 mg protein/g glucan. This improved saccharification of pretreated poplar with such low enzyme loadings can be attributed to the relatively high lignin removal during the pretreatment process. Reduced enzyme loadings are vital for making the overall process more cost effective, and several studies have therefore sought to utilize reduced enzyme loadings for the enzymatic hydrolysis step of pretreated woody biomass. Koo et al. [40] and Kumar et al. [54] also demonstrated low enzyme loading during the hydrolysis of mixed hardwood chips and softwood pulp, respectively, following pressurized oxygen pretreatments. In addition, Kim et al. [55] and Yamamoto et al. [56] obtained high sugar yields from woody biomass utilizing low enzyme loadings with liquid hot water and sulfur dioxide-ethanol–water fractionation pretreatments, respectively.

**Fig. 5:**
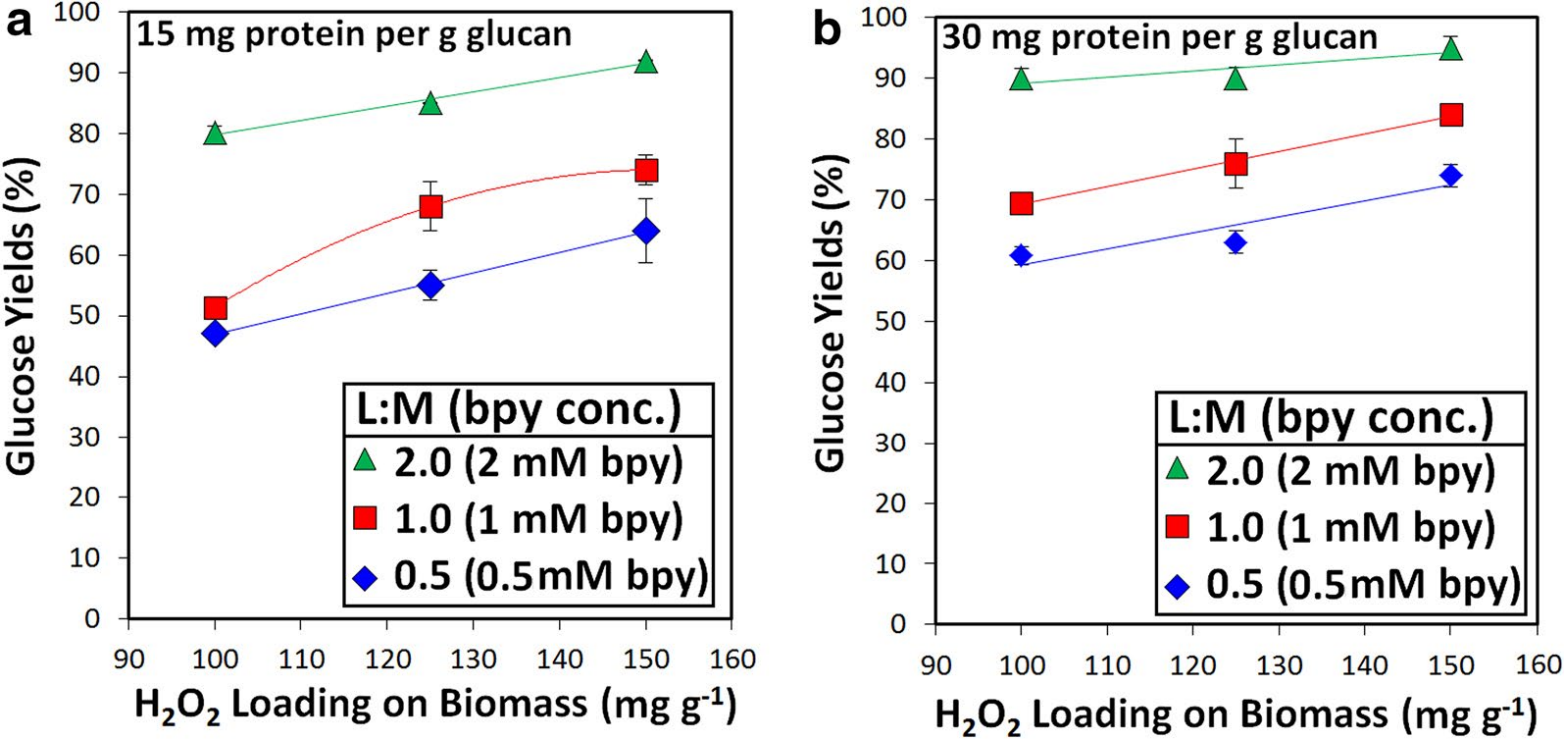
Impact of H_2_O_2_ loading and bipyridine concentration on glucose yields following alkaline pre-extraction and Cu-AHP pretreatment utilizing fed-batch addition of H_2_O_2_ for enzyme loadings of **a** 15 mg protein per g glucan and **b** 30 mg protein per g glucan. Pretreatment reactions were performed for 24 h at 10 % (w/v) solids. Final copper concentration in the reaction was 1 mM (5 μmol/g biomass). The data points are the averages of three independent experiments, and the error bars represent ± standard deviations of the means

In the third set of experiments, the impacts of the Cu:bpy ratio as well as the enzyme and H_2_O_2_ loadings on hydrolysis yields were determined for the fed-batch Cu-AHP process following alkaline pre-extraction. Figure 5 depicts sugar yields obtained at a catalyst concentration of 1 mM copper with varying ligand:metal ratios, H_2_O_2_ loadings, and enzyme loadings. These results reveal the potential for reduced loadings of costly ligand even at low enzyme loadings of 15 and 30 mg/g glucan. Glucose hydrolysis yields as high as 75 % were obtained at a ligand concentration of 0.5 mM, 30 mg/g glucan enzyme, and 150 mg H_2_O_2_. An increase in the glucose yields to 85 % was obtained by increasing the bipyridine concentration to 1.0 mM with 30 mg/g glucan enzyme and 150 mg H_2_O_2_. These results demonstrate the potential of the two-stage pretreatment approach coupled with fed-batch H_2_O_2_ addition to decrease bipyridine loadings.

Although promising, there is a substantial opportunity for process optimization to decrease further these input costs. Potential approaches include (a) decreasing the loading of the process input chemicals and/or enzymes, (b) improved recovery/regeneration of the process input chemicals, and (c) replacement of the process input chemicals with lower cost substitutes. An additional strategy is to optimize the alkaline pre-extraction for the delignification of wood chips, as delignification is known to lower substantially the energy requirements for particle-size reduction [57]. Furthermore, more severe alkaline pre-extraction conditions could be employed which would be expected to both decrease the second-stage treatment requirements and the required enzyme loadings to achieve target hydrolysis yields.

### Characterization of Cu-AHP solubilized lignin

**Fig. 6:**
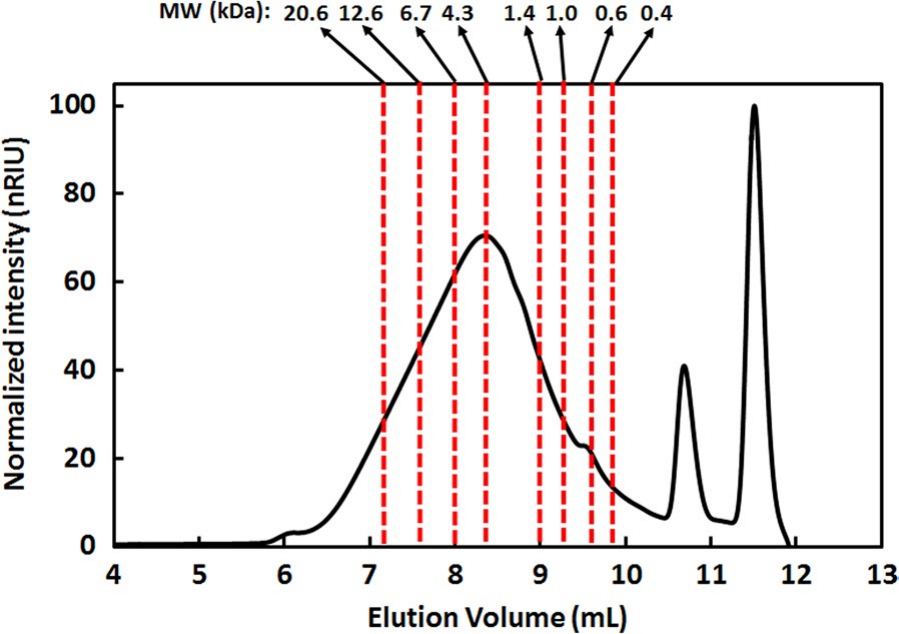
Size-exclusion chromatography elution profile for the lignin solubilized during Cu-AHP pretreatment with alkaline pre-extraction and fed-batch addition of H_2_O_2_

As shown in Fig. 3, the improved pretreatment strategy is capable of solubilizing a significant fraction of the total lignin present in the hybrid poplar. Recognizing that this delignification process not only improves the enzymatic digestibility of the biomass but also provides a lignin stream for potential valorization to chemicals and/or fuels, we sought to characterize the solubilized lignin. The solubilized lignin obtained from the two-stage, fed-batch Cu-AHP pretreatment was precipitated at pH 2, washed with water, and lyophilized to dryness. Compositional analysis of the recovered product (85 % of the total solubilized lignin) indicated that it was relatively pure, containing ≥90 % Klason lignin and only 3 % xylan and 2 % ash. Size-exclusion chromatography and two-dimensional heteronuclear single-quantum coherence (2D HSQC) NMR were applied for qualitative analyses of the lignin. The weight average (M_w_) and number average (M_n_) molecular weights of the lignin were found to be approximately 22,000 and 8000 Da, respectively (Fig. 6). This corresponds to an average of approximately 43 monomeric units per lignin polymer assuming a molar mass of 187 g/mol per lignin monomer. Thus, the lignin obtained after the improved AHP process was of high molecular weight which may assist in its further utilization for value-added products.

We also performed 2D HSQC NMR to ascertain the changes to the chemical structure of the solubilized lignin following the Cu-AHP pretreatment procedure (Additional file 1: Figure S3). Analysis of the spectra obtained for the solubilized lignin indicated the presence of small amounts of oxidized syringyl and guaiacyl units. Interestingly, there was no significant difference in the extent of lignin oxidation by the improved Cu-AHP process (i.e., alkaline extraction of the poplar followed by fed-batch Cu-AHP) compared to the reference case Cu-AHP [58]. Therefore, not only does the improved Cu-AHP process provide higher sugar yields and increased lignin solubilization, but it also preserves the lignin for subsequent valorization.

## Conclusions

Two different strategies were employed to improve Cu-AHP pretreatment of hybrid poplar. First, an alkaline extraction step, which increased biomass porosity and removed extractives that inhibit enzymatic hydrolysis, was introduced prior to Cu-AHP. This modification increased glucose yields from 63 to 86 %. In a second strategy, the H_2_O_2_ was utilized more efficiently by employing a fed-batch system, which resulted in an increase in glucose yields from 63 % in the unmodified Cu-AHP process to 77 %. Significantly, combining these two strategies (i.e., alkaline extraction followed by fed-batch addition of H_2_O_2_ during Cu-AHP) led to glucose and xylose yields that were 96 and 94 %, respectively, of theoretical maximum. In addition, clean and relatively unmodified lignin (~50 % of the original lignin) that is suitable for valorization was also generated. Importantly, experiments performed with different H_2_O_2_, catalyst, and enzyme loadings demonstrated the potential for further reduction of chemical inputs using this improved Cu-AHP pretreatment process.

## Methods

### Biomass and compositional analysis

Hybrid poplar (*Populus nigra* var. *charkoviensis* x *caudina* cv. NE-19) grown at the University of Wisconsin Arlington Agricultural Research Station was milled to pass through a 20-mesh screen (Thomas Scientific—3383L10—Mini-Mill, Thomas-Wiley). The initial compositions of the structural carbohydrates and the acid-insoluble lignin (Klason lignin) were determined using the National Renewable Energy Laboratory (NREL) two-stage acidolysis method [59]. Due to the inability of the Aminex HPX-87H column to resolve mannose and galactose from xylose, the xylose yields and contents reported using this method include mannose and galactose.

### Catalytic copper-catalyzed AHP pretreatment (reference case)

Biomass (0.51 g; ~0.50 g dry basis; approximately 3 % moisture content) was pretreated in a total of 5.0 mL aqueous solution (10 % solids loading). The reference case Cu-AHP pretreatment reaction was carried out by adding 4.33 mL of distilled water followed by 270 μL of 5 M NaOH (100 mg/g biomass), 125 μL of a 40 mM CuSO_4_ solution, 125 μL of a solution containing both 40 mM CuSO_4_ and 160 mM 2,2ˊ-bipyridine (bpy) (2 mM Cu^2+^ and 4 mM bpy final concentration), and finally 150 μL of 30 % H_2_O_2_ (w/w) (100 mg H_2_O_2_ per g biomass; 10 % loading) to the biomass. The reactants were briefly vortexed, and the slurry was incubated with orbital shaking at 180 rpm and 30 °C for 24 h. The initial pH for the Cu-AHP pretreatment reaction was approximately 11.5.

### Cu-AHP with fed-batch addition of hydrogen peroxide

Cu-AHP pretreatment with fed-batch addition of H_2_O_2_ was performed as described above for the reference case except that the 150 μL of 30 % H_2_O_2_ (w/w) (100 mg/g biomass final loading) was added to the reaction mixture in small batches over a 10-h period. Specifically, each hour 15 μL of 30 % H_2_O_2_ was added to the reaction mixture followed by a brief vortex to ensure an even distribution. Following the final addition of H_2_O_2_, the mixture was incubated as described for an additional 14 h (24 h total reaction time).

### Alkaline pre-extraction with Cu-AHP pretreatment

Alkaline pre-extracted hybrid poplar was prepared by incubating 0.51 g (~0.50 g dry basis) of biomass in 5 mL (10 % solids loading) of 270 mM NaOH(*aq*) (100 mg/g biomass final NaOH loading) at 30 °C for 1 h. After 1 h of incubation, the remaining insoluble biomass was washed with one volume of deionized water and subjected to 23 h of Cu-AHP pretreatment (24 h total reaction time including the 1-h pretreatment) as described above.

### Combined alkaline pre-extraction and Cu-AHP using fed-batch H_2_O_2_ addition

Alkaline pre-extraction and fed-batch H_2_O_2_ addition strategies were combined in the modified Cu-AHP pretreatment. Hybrid poplar biomass (0.51 g or ~0.50 g dry weight) was subjected to alkaline pre-extraction, washed with one volume of deionized water, and pretreated with Cu-AHP using fed-batch addition of H_2_O_2_ as described above except that the concentration of the Cu(bpy) complexes was reduced by 50 % (i.e., to 1.0 mM Cu^2+^ and 2.0 mM bpy total final concentrations). Note that whenever a different concentration of any of the reactants was used to probe the effect on the pretreatment process, an appropriate amount of distilled water was added to the reaction mixture to maintain a final solid biomass loading of 10 %.

### Enzymatic hydrolysis

Following pretreatment, the reaction mixture (5 mL aqueous plus ~0.5 g biomass) was combined with 0.5 mL of 1 M citric acid buffer (pH 5.0) and 4.3 mL of deionized water, and the slurry was slowly titrated with 72 % (w/w) H_2_SO_4_ to adjust the pH to 5.0 prior to enzymatic hydrolysis. An enzyme cocktail consisting of Cellic CTec3 and HTec3 (kindly provided by Novozymes A/S, Bagsværd, DK) at a loading of 30 mg protein/g glucan each unless otherwise noted was added to the hydrolysis reaction. The total aqueous volume of the reaction was then adjusted to 10 mL (5 % solids loading) by the addition of ~100 uL deionized water, and the samples were incubated at 50 °C for 72 h with orbital shaking at 210 rpm. Following enzymatic hydrolysis, the reaction contents were centrifuged, the pH was measured again to ensure there was no drift, and the amount of glucose and xylose in the supernatant was quantified by high-performance liquid chromatography (HPLC) (Agilent 1260 Series equipped with an infinity refractive index detector) using an Aminex HPX-87H column operating at 65 °C, a mobile phase of 5.0 mM H_2_SO_4_, and a flow rate of 0.6 mL/min. Standard curves using pure glucose and xylose were prepared prior to each analysis to convert peak area to concentration of monomeric sugar. The sugar yields (glucose and xylose) were calculated by dividing the amount of released sugar by the total sugar content of the biomass prior to pretreatment. The error bars in the figures represent the standard deviation from three or more biological replicates.

### Water retention value (WRV)

WRV was determined according to a modified version of TAPPI UM 256 [45]. After alkaline pre-extraction, the biomass (2 g) was washed with deionized water and vacuum-filtered to a moisture content of approximately 80 %. The washed wet biomass was inserted into a spin column (Handee Spin Column Cs4, Thermo Scientific) modified to have a 200 mesh stainless steel screen as the membrane directly under the biomass. The spin columns were then centrifuged at 900*g* for 15 min. After centrifugation, the biomass was weighed and placed in an aluminum tray at 105 °C for 3 h. The WRV value was calculated as the ratio of the mass of water retained by the wet biomass after centrifugation to the oven dry mass of the same biomass sample.

### Enzyme inhibition studies

Avicel (0.2 g) was mixed with 2.5 mL of the alkaline extraction liquor used to perform a prior pre-extraction reaction, followed by the addition of 0.25 mL of 1 M citric acid buffer (pH 5.0) and 2 mL of deionized water. The slurry was slowly titrated with 72 % (w/w) H_2_SO_4_ to adjust the pH to 5.0 prior to enzymatic hydrolysis. Following the addition of Cellic CTec3 at a loading of 30 mg protein/g glucan, the total aqueous volume of the enzymatic hydrolysis reaction was adjusted to 5 mL by the addition of deionized water (4 % solids loading), and the samples were incubated at 50 °C for 72 h with orbital shaking at 210 rpm. The pH was measured again at the end of hydrolysis to ensure that it was maintained at ~5.0.

### Lignin preparation

Following Cu-AHP pretreatment, the liquid phase was separated from the solid phase via filtration and the filtrate was acidified to pH 2.0 with 72 % (w/w) sulfuric acid. The precipitate was recovered by filtration, washed with aqueous sulfuric acid (pH 2.0), and finally washed by resuspending in deionized water. The suspension was centrifuged to remove the liquid phase. The precipitate was collected and lyophilized prior to analysis via size-exclusion chromatography and 2D HSQC NMR. Compositional analysis of lyophilized lignin was performed using the NREL two-stage acidolytic method [59].

### Lignin characterization

To analyze the lignin via size-exclusion chromatography, the lyophilized lignin samples were dissolved in a 80:20 (v/v) solution of 0.1 M NaNO_3_:0.005 M NaOH/CH_3_CN, syringe-filtered (0.22 μm), and loaded onto an Agilent 1260 series HPLC equipped with a Waters Ultrahydrogel ™ 250 (Milford, MA, USA) column. The mobile phase was also an 80:20 (v/v) mixture of 0.1 M NaNO_3_:0.005 M NaOH/CH_3_CN at a flow rate of 0.6 mL min^−1^ at 45 °C. The lignin was detected using an Agilent 1260 infinity refractive index detector (RID) detector. Polyethylene glycol (PEG) standards (0.4, 0.6, 1.0, 1.4, 4.3, 6.7, 12.6, and 20.6 kDa; Waters™) were run on the system to generate a standard curve. Based on the reference elution volumes, the number average (M_n_) and weight average (M_w_) molecular weights were calculated. The number of monomeric units was calculated by utilizing an approximate molecular weight of a lignin monomer as ~187 g/mol. 2D HSQC NMR was performed on the solubilized lignin according to the procedure outlined in Li et al. [58].

## Additional file

10.1186/s13068-016-0442-0 **Table S1.** Sugar yields obtained from different pretreatment strategies. **Table S2.** Percent mass loss obtained after different pretreatments. **Figure S1.** Water retention value (WRV) for untreated biomass and alkaline pre-extracted biomass. **Figure S2.** Enzymatic hydrolysis inhibition studies using Avicel as a substrate. **Figure S3.** Partial 2D HSQC NMR spectra of (A) lignin from untreated, debarked whole poplar, (B) solubilized lignin from reference case Cu-AHP pretreated poplar, and (C) solubilized lignin from modified Cu-AHP pretreated poplar.

## Abbreviations

AHP: alkaline hydrogen peroxide
AIR: alcohol insoluble residue
bpy: 2,2′-bipyridine
Cu-AHP: copper-catalyzed alkaline hydrogen peroxide
Da: dalton
HPLC: high-performance liquid chromatography
HSQC: heteronuclear single-quantum coherence
M_n_: number average molecular weight
M_w_: weight average molecular weight
NMR: nuclear magnetic resonance
NREL: National Renewable Energy Laboratory
PEG: polyethylene glycol
RID: refractive index detector
TAPPI: Technical Association of the Pulp and Paper Industry
WRV: water retention value
