# Supplementary material for: Effective alkaline metal-catalyzed oxidative delignification of hybrid poplar
Source: Biotechnol Biofuels. 2016 Feb 9;9:34. doi: 10.1186/s13068-016-0442-0 (PMC4746924; doi:10.1186/s13068-016-0442-0)
Supplement: Supplementary file 1 — 10.1186/s13068-016-0442-0 Table S1. Sugar yields obtained from different pretreatment strategies. Table S2. Percent mass loss obtained after different pretreatments. Figure S1. Water retention value (WRV) for untreated biomass and alkaline pre-extracted biomass. Figure S2. Enzymatic hydrolysis inhibition studies using Avicel as a substrate. Figure S3. Partial 2D HSQC NMR spectra of (A) lignin from untreated, debarked whole poplar, (B) solubilized lignin from reference case Cu-AHP pretreated poplar, and (C) solubilized lignin from modified Cu-AHP pretreated poplar. [file 13068_2016_442_MOESM1_ESM.pdf]

Supplementary Material

for

Effective alkaline metal-catalyzed oxidative delignification of hybrid poplar

Aditya Bhalla[1,2], Namita Bansal[1,2], Ryan J. Stoklosa[1,3], Mackenzie Fountain[2], John Ralph[4], David B. Hodge[1,3,5]*, Eric L. Hegg[1,2]*

[1] DOE Great Lakes Bioenergy Research Center, Michigan State University, USA

[2] Department of Biochemistry & Molecular Biology, Michigan State University USA

[3] Department of Chemical Engineering & Materials Science, Michigan State University, USA

[4] DOE Great Lakes Bioenergy Research Center, University of Wisconsin-Madison, USA

[5] Division of Sustainable Process Engineering, Luleå University of Technology, Sweden

Table S1: Sugar yields obtained from different pretreatment strategies

	**Glucose yields (%)**	**Xylose yields (%)**
Cu-AHP reference case	62.8±1.5	73.8±1.7
Alkaline pre-extraction + Cu-AHP	86.0±0.7	95.4±1.2
Cu-AHP with fed-batch addition of H_2O_2	77.0±0.3	92.6±0.4
Alkaline pre-extraction + fed-batch Cu-AHP	96.2±0.6	93.9±2.5

The values reported are the averages of three biological replicates, and the error bars indicate ± standard deviations of the means.

Table S2: Percent mass loss obtained after different pretreatments

**Pretreatments of hybrid poplar**	**Glucan loss (%)**	**Xylan loss (%)**	**Lignin loss (%)**
Untreated	N/A	N/A	N/A
Cu-AHP reference case	3.0±0.1	22.6±0.3	28.0±0.4
Alkaline pre-extraction	0.6±1.2	4.9±0.3	5.0±0.4
Alkaline pre-extraction + Cu-AHP	4.8±1.5	31.6±0.3	40.0±1.4
Cu-AHP with fed-batch addition of H_2O_2	6.2±0.9	22.5±0.6	44.0±0.0
Alkaline pre-extraction + fed-batch Cu-AHP	5.8±0.4	39.1±0.3	56.0±0.3

The values reported are the averages of three biological replicates, and the error bars indicate ± standard deviations of the means.

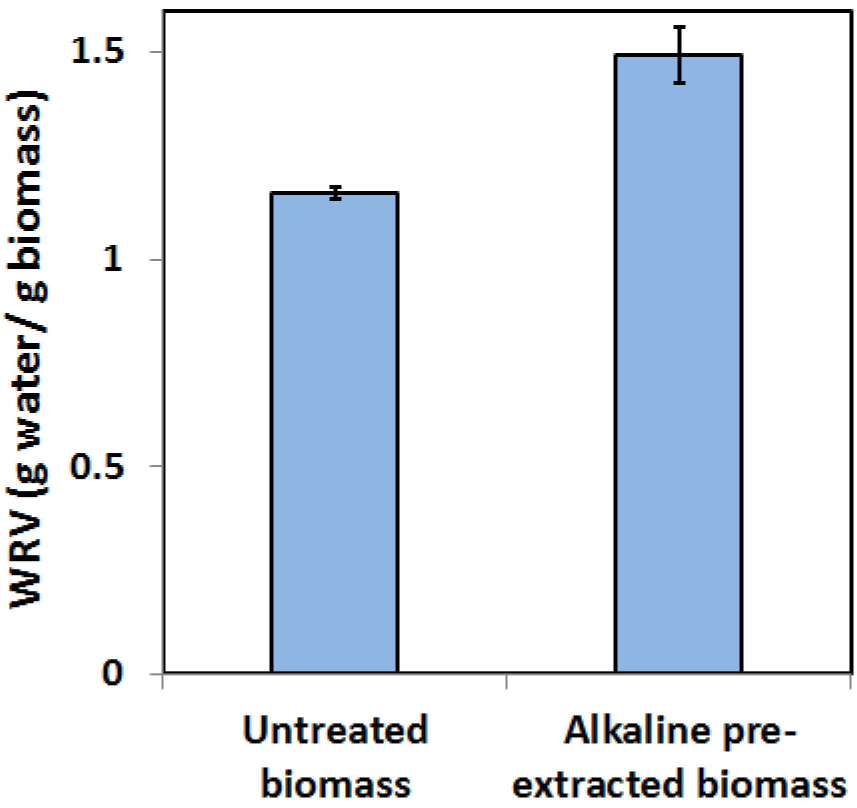

Figure S1: Water retention value (WRV) for untreated biomass and alkaline pre-extracted biomass. The values reported are the averages of three biological replicates, and the error bars indicate ± standard deviations of the means.

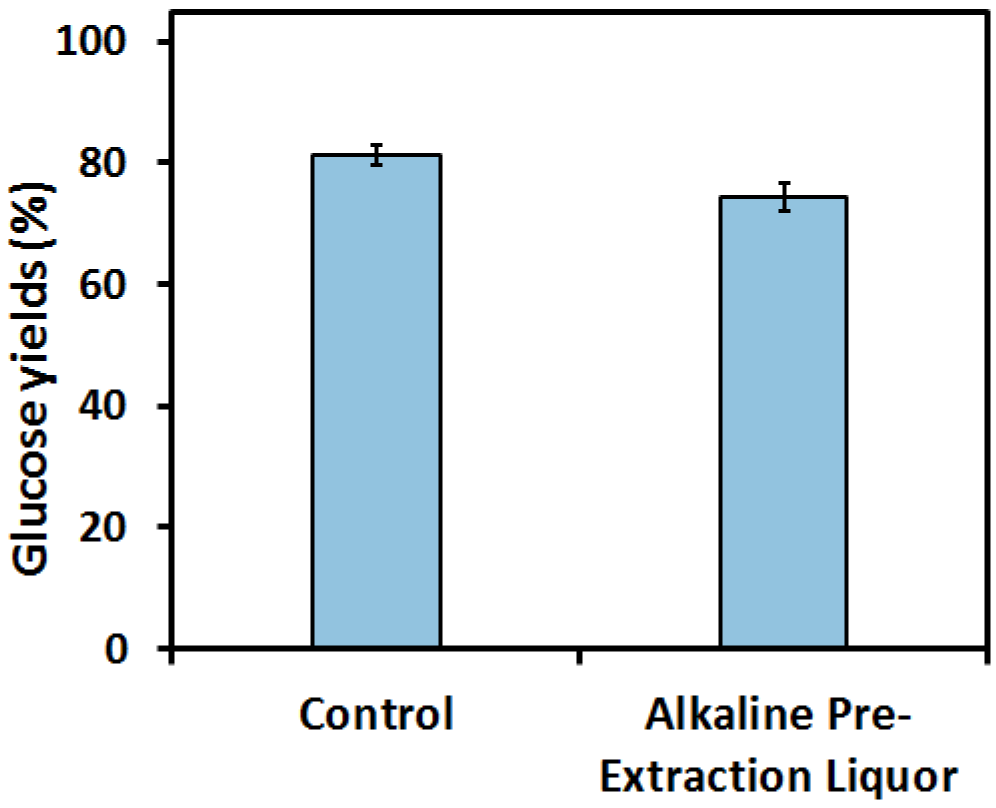

Figure S2. Enzymatic hydrolysis inhibition studies using Avicel as a substrate. Control: Water added as the inhibitor; Alkaline Pre-extraction liquor: Liquor obtained after alkaline pre-extraction of hybrid poplar added as the inhibitor. The values reported are the averages of the three biological replicates, and the error bars indicate ± standard deviations of the means.

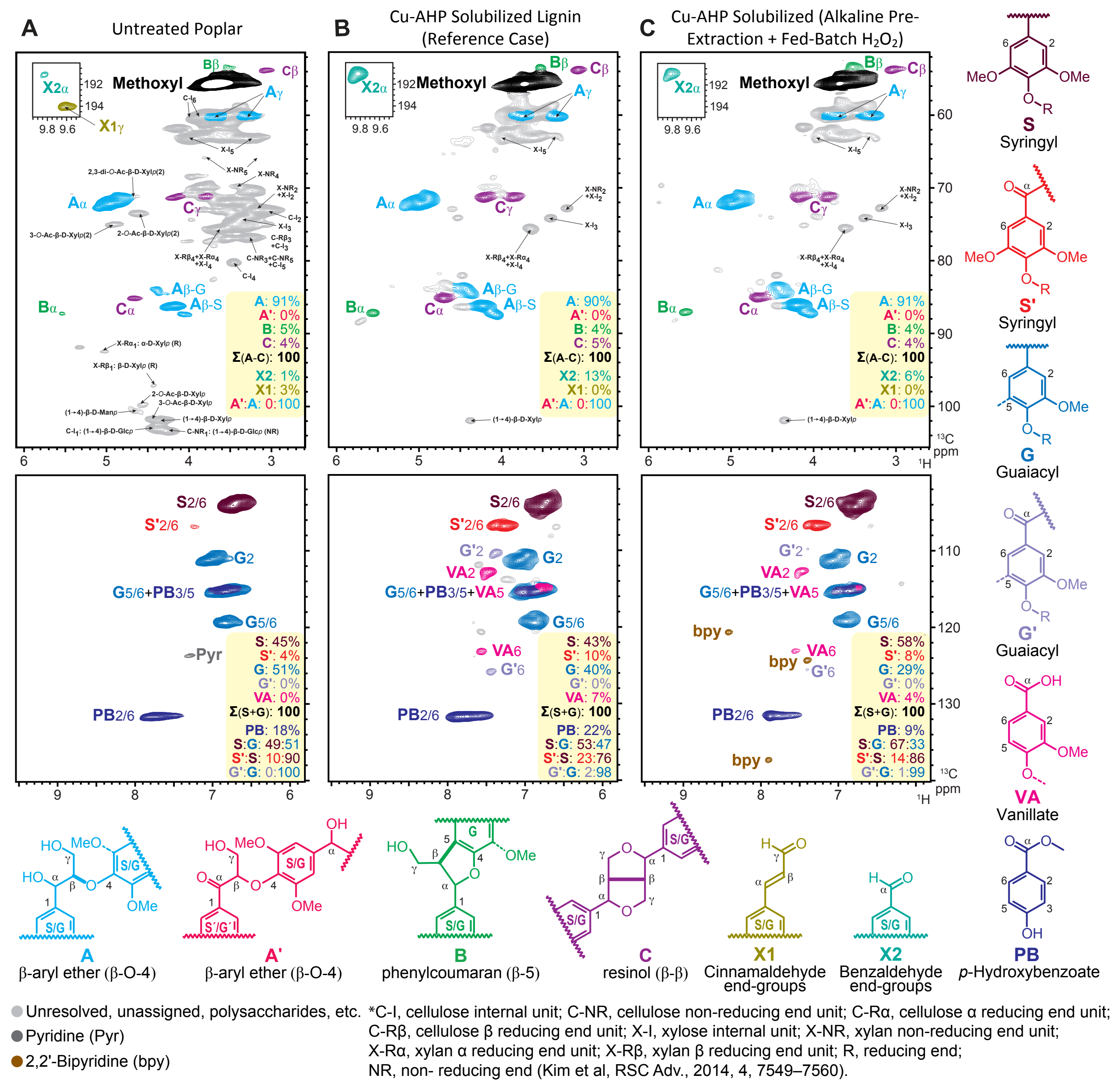

A
Untreated Poplar
B
Cu-AHP Solubilized Lignin (Reference Case)
C
Cu-AHP Solubilized (Alkaline Pre-Extraction + Fed-Batch H2O2)
Methoxyl
X2α
X1γ
Bβ
Cβ
Aγ
Aα
Cγ
Aβ-G
Aβ-S
Bα
Cα
A: 91%
A': 0%
B: 5%
C: 4%
Σ(A-C): 100
X2: 1%
X1: 3%
A':A: 0:100
A: 90%
A': 0%
B: 4%
C: 5%
Σ(A-C): 100
X2: 13%
X1: 0%
A':A: 0:100
A: 91%
A': 0%
B: 4%
C: 4%
Σ(A-C): 100
X2: 6%
X1: 0%
A':A: 0:100
S2/6
S'2/6
G2
G'2
VA2
G5/6+PB3/5
G5/6+PB3/5+VA5
G5/6
VA6
G'6
Pyr
bpy
PB2/6
S: 45%
S': 4%
G: 51%
G': 0%
VA: 0%
Σ(S+G): 100
PB: 18%
S:G: 49:51
S':S: 10:90
G':G: 0:100
S: 43%
S': 10%
G: 40%
G': 0%
VA: 7%
Σ(S+G): 100
PB: 22%
S:G: 53:47
S':S: 23:76
G':G: 2:98
S: 58%
S': 8%
G: 29%
G': 0%
VA: 4%
Σ(S+G): 100
PB: 9%
S:G: 67:33
S':S: 14:86
G':G: 1:99
13C ppm
1H
S Syringyl
S' Syringyl
G Guaiacyl
G' Guaiacyl
VA Vanillate
A β-aryl ether (β-O-4)
A' β-aryl ether (β-O-4)
B phenylcoumaran (β-5)
C resinol (β-β)
X1 Cinnamaldehyde end-groups
X2 Benzaldehyde end-groups
PB p-Hydroxybenzoate
Unresolved, unassigned, polysaccharides, etc.
Pyridine (Pyr)
2,2'-Bipyridine (bpy)
*C-I, cellulose internal unit; C-NR, cellulose non-reducing end unit; C-Rα, cellulose α reducing end unit; C-Rβ, cellulose β reducing end unit; X-I, xylose internal unit; X-NR, xylan non-reducing end unit; X-Rα, xylan α reducing end unit; X-Rβ, xylan β reducing end unit; R, reducing end; NR, non- reducing end (Kim et al, RSC Adv., 2014, 4, 7549–7560).

Figure S3. Partial 2D HSQC NMR spectra of (A) lignin from untreated, debarked whole poplar, (B) solubilized lignin from reference case Cu-AHP pretreated poplar, and (C) solubilized lignin from modified Cu-AHP pretreated poplar. Upper panels depict the aliphatic region and the lower panels depict the aromatic region. Contours are colored to match the structures.
